# Combined Visualization of Nigrosome-1 and Neuromelanin in the Substantia Nigra Using 3T MRI for the Differential Diagnosis of Essential Tremor and *de novo* Parkinson's Disease

**DOI:** 10.3389/fneur.2019.00100

**Published:** 2019-02-12

**Authors:** Lirong Jin, Jian Wang, Changpeng Wang, Danlan Lian, Ying Zhou, Yong Zhang, Minzhi Lv, Yuanfang Li, Zhen Huang, Xiaoqin Cheng, Guoqiang Fei, Kai Liu, Mengsu Zeng, Chunjiu Zhong

**Affiliations:** ^1^Department of Neurology, Zhongshan Hospital, Fudan University, Shanghai, China; ^2^Department of Radiology, Zhongshan Hospital, Fudan University, Shanghai, China; ^3^Shanghai Medical Imaging Institute, Shanghai, China; ^4^Department of Radiology, Xiamen Branch, Zhongshan Hospital, Fudan University, Xiamen, China; ^5^Department of Neurology, Xiamen Branch, Zhongshan Hospital, Fudan University, Xiamen, China; ^6^MR Research, GE Healthcare, Shanghai, China; ^7^Department of Biostatistics, Zhongshan Hospital, Fudan University, Shanghai, China

**Keywords:** Parkinson's disease, essential tremor, neuromelanin, nigrosome-1, QSM, substantia nigra

## Abstract

Differentiating early-stage Parkinson's disease (PD) from essential tremor (ET) remains challenging. In the current study, we aimed to evaluate whether visual analyses of neuromelanin-sensitive magnetic resonance imaging (NM-MRI) combined with nigrosome-1 (N1) imaging using quantitative susceptibility mapping (QSM) in the substantia nigra (SN) are of diagnostic value in the differentiation of *de novo* PD from untreated ET. Sixty-eight patients with *de novo* PD, 25 patients with untreated ET, and 34 control participants underwent NM-MRI and QSM. NM and N1 signals in the SN on MR images were visually evaluated using a 3-point ordinal scale. Receiver operating characteristic (ROC) analyses were performed to determine the diagnostic values of the visual ratings of NM and N1. The diagnostic values of the predicted probabilities were calculated via logistic regression analysis using the combination of NM and N1 visual ratings, as well as their quadratic items. The proportions of invisible NM and invisible N1 were significantly higher in the PD group than those in the ET and control groups (*p* < 0.001). The sensitivity/specificity for differentiating PD from ET was 0.882/0.800 for NM and 0.794/0.920 for N1, respectively. Combining the two biomarkers, the area under the curve (AUC) of the predicted probabilities was 0.935, and the sensitivity/specificity was 0.853/0.920 when the cutoff value was set to 0.704. Our findings demonstrate that visual analyses combing NM and N1 imaging in the SN may aid in differential diagnosis of PD and ET. Furthermore, our results suggest that patients with PD exhibit larger iron deposits in the SN than those with ET.

## Introduction

Parkinson's disease (PD) and essential tremor (ET) are common movement disorders, especially among older adults (1). PD is characterized by motor symptoms including bradykinesia, resting tremor, and rigidity, while ET often manifests as isolated tremor in the bilateral upper limbs. Although they are distinct entities, these two movement disorders may share some clinical characteristics, such as non-motor features and resting/postural tremor, as well as genetic and pathological mechanisms (2, 3). Hence, the differentiation of PD and ET remains challenging, especially early in the disease course.

Neuroimaging may aid in the differentiation of the two movement disorders. Dopamine transporter (DAT) imaging of the striatum is recommended in the differential diagnosis of PD (4). However, DAT imaging is expensive, subjects the patient to low doses of radiation, and is only available in specialized centers, limiting its clinical application. In contrast, MRI is widely available and does not subject the patient to radiation. Recent studies have provided evidence that MRI biomarkers may aid in the diagnosis of movement disorders (5, 6) and help to reveal the pathological changes correlated with motor and non-motor symptoms (7).

The pathological hallmarks of PD include progressive neurodegeneration of dopaminergic neurons and iron overload in substantia nigra (SN) (8). In contrast, whether ET is a degenerative disease is still debated, although some studies suggest that it is associated with cerebellar degeneration (9). Dopaminergic neurons in the SN contain a black pigment called neuromelanin (NM). Based on the paramagnetic properties of NM, high-resolution T1-weighted fast spin echo (FSE) imaging at high field strength (e.g., 3T) can visualize NM-generated contrast. This technique is referred to as neuromelanin-sensitive MRI (NM-MRI) (10). Previous studies have indicated that the signal intensity of NM is decreased in patients with PD (10, 11), even in the early stage of the disease (12), while it remains unchanged in patients with ET (13, 14), when compared with that in healthy controls. In addition, nigrosome-1 (N1), the largest of the five described nigrosomes, is most affected in patients with PD (~98% neuronal loss in N1) (15). N1 represents the pockets of high signal intensity in the dorsal part of the healthy SN, at intermediate and caudal levels on high resolution T2^*^/SWI, and can be visualized as a “swallow-tail sign” (16, 17). However, hyperintensity of N1 is absent in most patients with PD (17), possibly due to increases in iron deposition that occur in parallel to the loss of dopaminergic cells (16). Quantitative susceptibility mapping (QSM) may overcome several non-local restrictions of SWI and phase imaging, allowing for the quantification of iron content (18, 19). Indeed, this method may be more sensitive for detecting iron-related changes in patients with PD (20). To our knowledge, however, no studies have investigated N1 appearance in patients with ET.

Unlike voxel-based morphometry, diffusion tensor imaging, or blood oxygenation level-dependent imaging, which require complicated post-processing or quantitative measurements, NM on NM-MRI and N1 on QSM can be assessed visually, making these methods feasible for clinical application. To the best of our knowledge, no previous studies have investigated the combination of these two MR sequences for the differential diagnosis of PD and ET. In the current study, we aimed to evaluate whether visual analyses of NM imaging using NM-MRI combined with N1 imaging using QSM in the SN are of diagnostic value in the differentiation of *de novo* PD from untreated ET.

## Participants and Methods

### Participants

Sixty-eight patients with *de novo* PD, 25 patients with untreated ET, and 34 healthy controls were voluntarily recruited between September 2016 and April 2018. Patients with PD were diagnosed in accordance with MDS clinical diagnostic criteria for PD (4), while patients with ET were diagnosed in accordance with the criteria outlined in the Consensus Statement of the Movement Disorders Society on Tremor (21), by two movement specialists (J.L.R. and H.Z.). All patients were drug naïve. All control participants were recruited as volunteers from the community and had no history of neurological/psychiatric disorders. Exclusion criteria were as follows: history of other neurological/psychiatric disorders, severe infection, liver dysfunction, renal insufficiency, past/current substance abuse, tremor-related dysmetabolism including thyroid dysfunction and drug toxicity, and abnormal signals that affected further analyses on structural MRI. Unified Parkinson's Disease Rating Scale (UPDRS) motor scores were obtained for all patients with PD and ET. This study was approved by the Committee on Medical Ethics of Zhongshan Hospital, Fudan University. Written informed consent was obtained from all participants.

### MRI Protocol and Imaging Analysis

#### Imaging Protocol

All MR images were acquired using a 3T MR unit (Discovery™ MR750, GE Healthcare, Milwaukee, WI). A T1-weighted fast spin-echo sequence was obtained for NM-MRI images, as previously described (22), and the imaging parameters were as follows: repetition time/echo time (TR/TE), 600/13 ms; echo-train length, 2; section thickness, 2.5 mm, with no intersection gap; number of slices, 16; matrix size, 512 × 320; field-of-view (FOV), 220 mm; NEX, 5. A three-dimensional multi-echo GRE sequence was used to acquire T2^*^-weighted images, and the scanning parameters were as follows: TR: 51.5 ms; number of echoes, 16; first TE, 2.9 ms; TE spacing, 3 ms; bandwidth, 62.50 kHz; flip angle, 12°; FOV, 22 cm; matrix, 220 × 220; slice thickness, 2 mm; acceleration factor, 2; slices, 66. Afterwards, the QSM images were reconstructed from T2^*^-weighted images as described in previous studies (20). In addition, conventional MRI sequences including T1-weighted images, T2-weighted fluid-attenuated inversion recovery (FLAIR) images, and diffusion-weighted images (DWI) were obtained to exclude other pathological imaging findings that may have interfered with further assessment. The axial sections were scanned parallel to the anterior commissure-posterior commissure line with whole-brain coverage for QSM and routine MRI scans, and with coverage from the posterior commissure to the pons for NM-MRI.

#### Visual Analysis of Imaging Data

**Figure 1:**
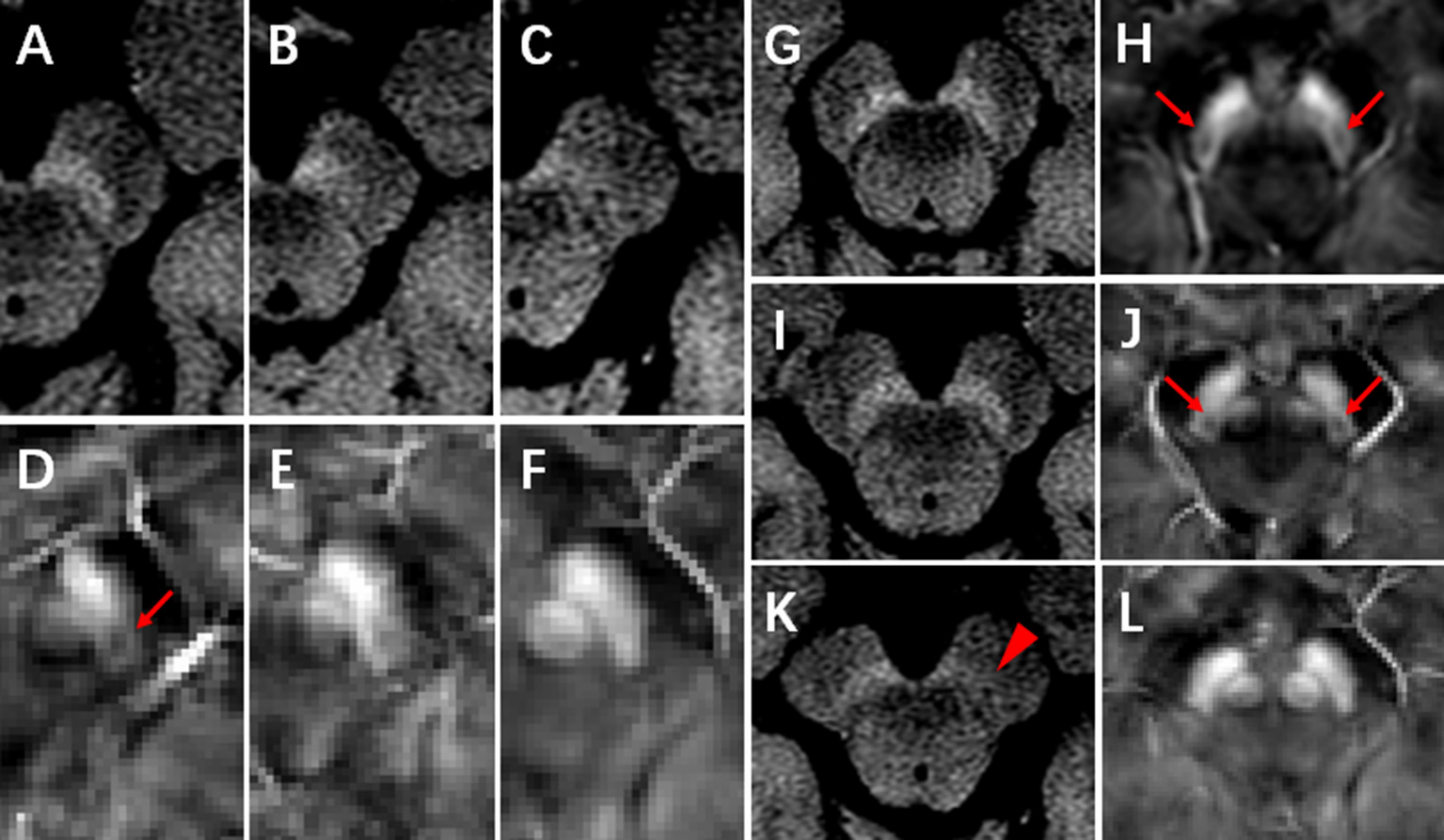
NM-MRI and QSM images. **(A–C)** represent the normal, possibly abnormal, and definitely abnormal SN on NM-MRI, respectively. Nigrosome-1 (N1) could be visualized in the dorsal part of the healthy SN on QSM images (**D**, arrow). **(D–F)** represent that N1 was present, indecisively present and absent, respectively. **(G,H)**, a control subject, female, 65 years, neuromelanin was normal **(G)** and N1 was present (**H**, arrow) in bilateral SN. **(I,J)**, an ET patient, 59 years, female, neuromelanin was normal **(I)** and N1 was present (**J**, arrow) in bilateral SN. **(K,L)**, a *de novo* PD patient, 75 years, female, neuromelanin was definitely abnormal in unilateral SN (**K**, arrowhead) and N1 was absent in bilateral SN **(L)**.

The NM-MR images were transferred to a workstation (ADW4.6, GE Healthcare) and displayed using certain settings (window width: 400, window level: 800–900) for analysis. The method for visual analysis was based on that described in a previous study (23), with modifications. We classified NM-MR images according to an 3-point ordinal scale, as follows: 0, normal view of the SN with high signal intensity bilaterally and no volume loss, indicating a healthy SN; 1, possible abnormality with reduced signal or volume of the SN unilaterally or bilaterally, indicating possible SN pathology; 2, definite abnormality with reduced signal or volume of the SN, indicating SN pathology (Figure 1).

QSM data were transferred to a local computer, and images were viewed using MRIcro software (Version: 1.40 build 1). N1 was visualized as an oval-shaped area of low signal intensity surrounded by hyperintensity on QSM in the dorsal part of the healthy SN, at intermediate and caudal levels. Visual analysis of N1 was performed using a 3-point ordinal scale, as follows: 0, normal, N1 present bilaterally; 1, non-diagnostic, indecisive presence of N1 unilaterally or bilaterally; 2, pathological, N1 absent bilaterally (Figure 1). Two radiologists (rater 1: W.J. and rater 2: L.D.L.), who were blinded to participants information, independently performed visual analyses twice, with an interval of at least 7 days. The intra- and inter-rater agreement of visual scores was determined using weighted kappa values. For the conflicting cases between the two raters, visual analyses were conducted by a third radiologist (with 30 years of experience) to acquire a final rating for statistical analyses.

### Statistical Analyses

The results were expressed as the mean ± SD. One-way analyses of variance (ANOVA), Mann-Whitney *U*-test, and chi-square test were used to compare demographic data. Logistic regression analyses were employed to estimate the combined predicted probabilities of the visual ratings for NM and N1. Diagnostic performances in visual assessments of NM and N1, separately, and in the predicted probabilities were analyzed using receiver operating characteristic (ROC) curves. The chi-square test was used to compare the area under the curve (AUC) values of the different diagnostic models. The level of significance was set to 0.05, and all tests were two-sided. Statistical analyses were performed using SPSS version 22.0 (SPSS Inc., Chicago IL) and Stata/SE version 14.0 (StataCorp LP).

## Results

### Demographic and Clinical Data

The demographic and clinical characteristics of all participants are summarized in Table 1. No significant differences in gender, age, Mini Mental State Examination (MMSE) scores, or Montreal Cognitive Assessment (MoCA) scores were observed among the three groups. Disease duration was significantly longer in patients with ET than in patients with PD (*p* < 0.001), although there was no significant difference in UPDRS tremor scores between the ET and PD groups. All patients with PD had mild disease severity (Hoehn and Yahr stages 1 to 2).

**Table 1:** Clinical data for patients with PD, patients with ET, and controls.

	**PD group**	**ET group**	**Control group**	***p*-value**
	**(*n* = 68)**	**(*n* = 25)**	**(*n* = 34)**	
Gender (M/F)	28/40	10/15	13/21	0.960
Age (years)	64.54 ± 9.67	61.12 ± 11.16	63.53 ± 7.81	0.311
MMSE score	27.75 ± 2.71	28.63 ± 1.86	28.80 ± 1.38	0.069
MoCA score	24.98 ± 3.75	25.83 ± 3.28	26.33 ± 2.28	0.165
Disease duration (years)	1.86 ± 1.68	8.60 ± 7.85		< 0.001^*^
UPDRSIII tremor scores	2.06 ± 1.74	3.67 ± 3.27		0.063

* *Significantly different; PD, Parkinson's Disease; ET, Essential Tremor; MMSE, Mini Mental State Examination; MoCA, Montreal Cognitive Assessment; UPDRS, United Parkinson's Disease Rating Scale*.

### Visual Analyses of the SN on NM-MRI and N1 on QSM

The proportion of conflicting cases was 22.8% (29/127) for NM-MRI analyses and 11.8% (15/127) for N1-QSM analyses for rater 1. For rater 2, the proportion of conflicting cases was 28.3% (36/127) for NM-MRI analyses and 22.0% (28/127) for N1-QSM analyses. The proportion of conflicting cases between the two observers was 25.2% (32/127) for NM-MRI analyses and 24.4% (31/127) for N1-QSM analyses. Thus, the weighted kappa coefficient was calculated to evaluate intra- and inter-rater agreement for visual analyses. For rater 1, intra-rater agreement values for NM and N1 were 0.837 and 0.903, respectively. For rater 2, intra-rater agreement values for NM and N1 were 0.828 and 0.815, respectively. Furthermore, the inter-rater agreement was 0.827 for NM and 0.777 for N1. Thus, visual analyses were highly consistent.

**Figure 2:**
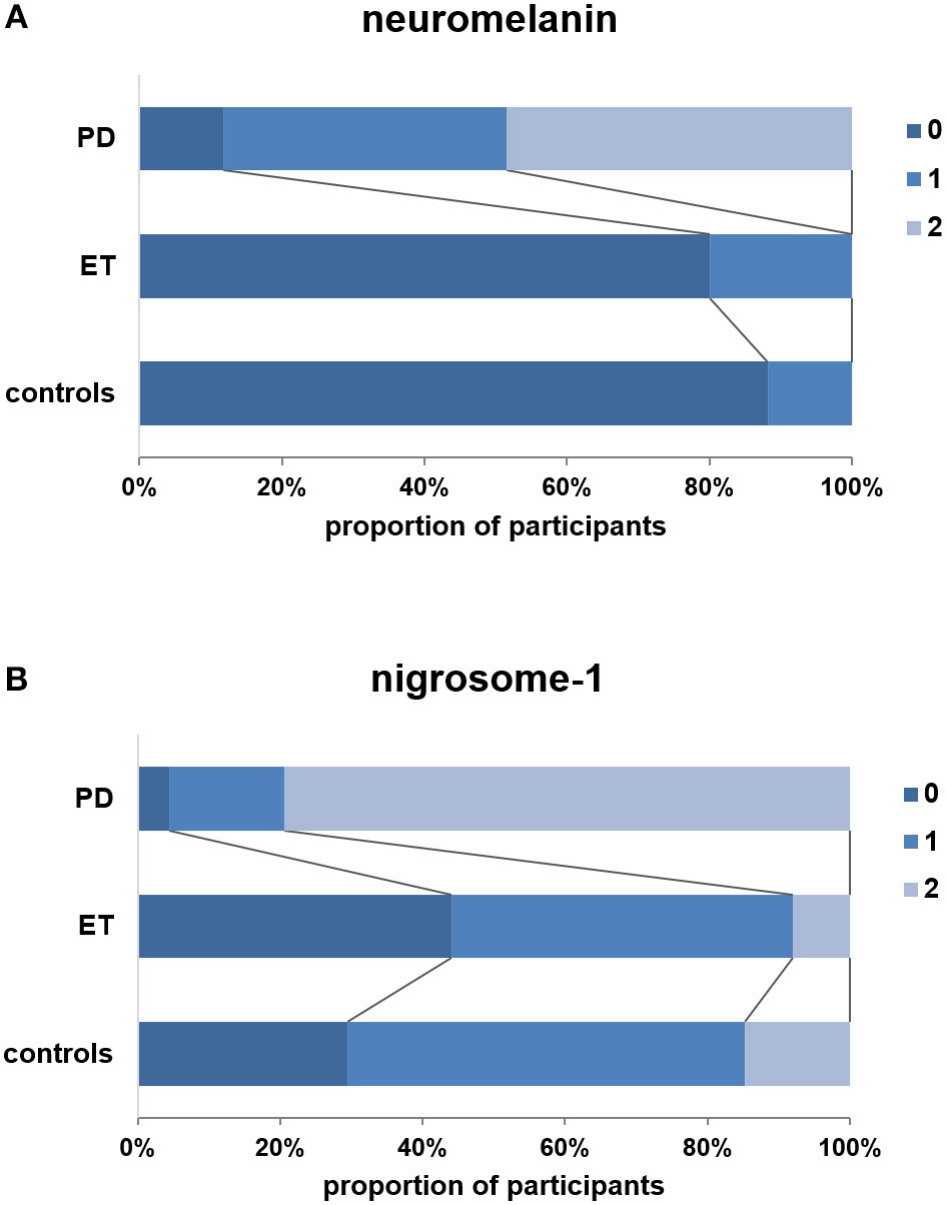
Visual scores (3-point ordinal scale) for patients with PD, patients with ET, and controls. **(A)** Visual scores for neuromelanin. **(B)** Visual scores for nigrosome-1. Note that no definitely abnormal neuromelanin signals (score: 2) were observed in the ET and control groups, and the frequency of nigrosome-1 absence (score: 2) was significantly lower in the ET group than in the PD group (*p* < 0.001).

For NM visual analysis, scores were 0 in 8 patients with PD (11.8%), 1 in 27 patients with PD (39.7%), and 2 in 33 patients with PD (48.5%). In the ET group, NM scores were 0 in 20 patients (80.0%) and 1 in 5 patients (20.0%). In the control group, NM scores were 0 in 30 participants (88.2%) and 1 in 4 participants (11.8%) (Figure 2A). NM scores of 2 were not observed in the ET and control groups, and the proportion of NM ratings did not significantly differ between these two groups (*p* = 0.385). However, the proportion of NM ratings in the PD group differed significantly from that in the ET group (*p* < 0.001).

N1 scores were 0 in 3 patients with PD (4.4%), 1 in 11 patients with PD (16.2%), and 2 in 54 patients with PD (79.4%). In the ET group, N1 scores were 0 in 11 patients (44.0%), 1 in 12 patients (48.0%), and 2 in 2 patients (8.0%). In the control group, N1 scores were 0 in 10 participants (29.4%), 1 in 19 participants (55.9%), and 2 in 5 participants (14.7%) (Figure 2B). The proportion of N1 ratings in the ET group did not significantly differ from that in controls (*p* = 0.454). However, the proportion of patients with N1 scores of 2 was significantly lower in the ET group than in the PD group (*p* < 0.001). Representative images obtained from patients with PD and ET and controls are presented in Figure 1.

### Diagnostic Performances of Visual Analyses of NM and N1, and of the Predicted Probabilities Combining the Two Biomarkers

Based on our findings, we then employed ROC analysis to assess the diagnostic values of several models for differentiating PD from ET. The AUC of NM-MRI (model 1) for differentiating PD from ET was 0.890 (95% CI 0.822, 0.958), and the sensitivity and specificity were 0.882 and 0.800, respectively, when the cutoff value for NM scores was set to ≥1. The AUC of N1 on QSM (model 2) for differentiating PD from ET was 0.882 (95% CI 0.802, 0.962), and the sensitivity and specificity were 0.794 and 0.920, respectively, when the cutoff value for N1 scores was set to 2. No significant differences in AUC values were observed between these two models (*p* > 0.05, Table 2).

**Table 2:** Diagnostic models for differentiating PD from ET.

	**AUC**	**Std. Err**.	**95% CI of AUC**	**Cutoff value**	**Sensitivity**	**Specificity**
Model 1^a^	0.890	0.035	0.822 0.958	≥1	0.882	0.800
Model 2^b^^#^	0.882	0.041	0.802 0.962	=2	0.794	0.920
Model 3^c^^§^	0.933	0.026	0.883 0.983	0.848	0.809	0.960
Model 4^d^^$^	0.935	0.026	0.884 0.986	0.704	0.853	0.920

a *Model 1: NM*.

b *Model 2: N1*.

# *No significant difference was observed between the AUCs of Model 1 and Model 2*.

c *Model 3: predicted probabilities. Log it = −2.176 + 1.923 × NM + 1.429 × N1*.

§ *The AUC of model 3 was significantly higher than that of Model 1*.

d *Model 4: predicted probabilities. Log it = −1.499 + 2.014 × NM – 1.194 × N1 + 1.267 × N1^2^*.

$ *The AUC of model 4 was significantly higher than those of Model 1 and Model 2*.

*PD, Parkinson's Disease; ET, Essential Tremor; NM, Neuromelanin; N1, Nigrosome-1; AUC, Area Under the Curve*.

We calculated the predicted probabilities using logistic regression to further explore the diagnostic performance of these two biomarkers. Model 3 was established using the predicted probabilities obtained by simply combining these two biomarkers (Log *it* = −2.176 + 1.923 × NM + 1.429 × N1). The AUC of model 3 was 0.933 (95% CI 0.883, 0.983). The sensitivity and specificity of model 3 were 0.809 and 0.960, respectively, when the cutoff value was set to 0.848. The AUC of model 3 was significantly higher than that of model 1 (*p* = 0.009), whereas it did not significantly differ from that of model 2 (*p* = 0.051, Table 2). Furthermore, the specificity of model 3 was higher than those of model 1 and 2 while the sensitivity of model 3 was lower than that of model 1.

**Figure 3:**
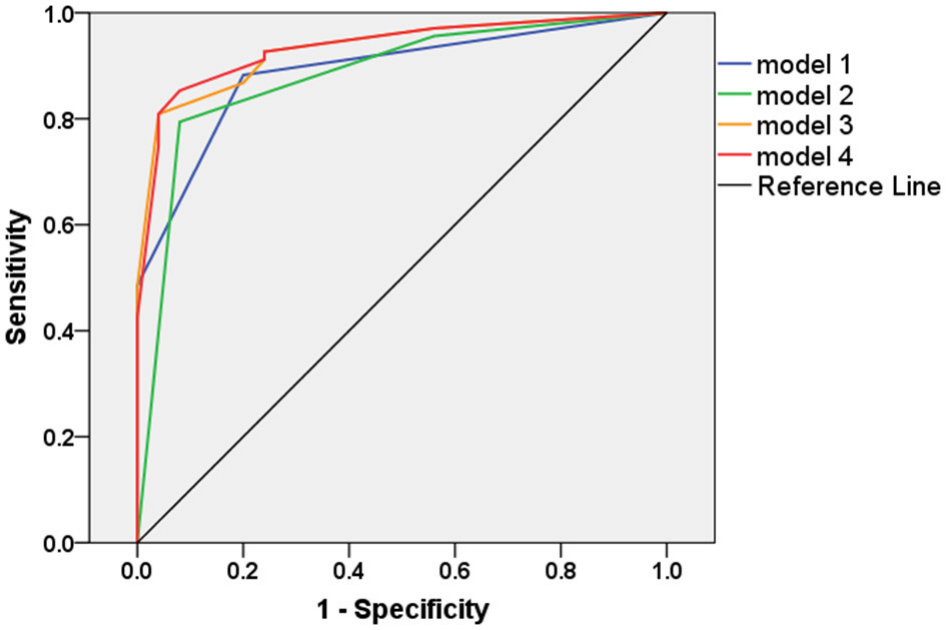
The receiver operating characteristic (ROC) curves of the four diagnostic models.

Model 4 was established using the predicted probabilities obtained by combining NM, N1, and N1^2^ (Log *it* = −1.499 + 2.014 × NM−1.194 × N1 + 1.267 × N1^2^). The AUC was 0.935 (95% CI 0.884, 0.986). The sensitivity and specificity of model 4 were 0.853 and 0.920, respectively, when the cutoff value was set to 0.704. The AUC of model 4 was significantly higher than those of model 1 (*p* = 0.041) and model 2 (*p* = 0.014). The sensitivity and specificity of model 4 were best among the four diagnostic models (Table 2). We further established model 5 using the predicted probabilities obtained by combining NM, N1, NM^2^, and N1^2^. However, the results of model 5 were not better than those of model 4 (data not shown). The ROCs of the four diagnostic models are shown in Figure 3.

## Discussion

In the current study, we aimed to evaluate whether visual analyses of NM and N1 imaging in the SN are of diagnostic value in the differentiation of *de novo* PD from untreated ET. Patients with PD exhibited reduced signal intensity on NM imaging and an absence of N1 in the SN, relative to patients with ET and healthy controls. Moreover, when visual analyses of NM and N1 imaging were combined, the model exhibited high diagnostic accuracy for differentiating *de novo* PD from untreated ET. To date, although ET is a common neurological disease, the pathogenic mechanisms remain poorly understood (24), and there are no adequate diagnostic biomarkers. Our findings suggest that non-invasive neuroimaging studies may aid in the differential diagnosis of tremor disorders, particularly PD and ET.

NM plays an important role in the pathogenesis of PD (25). Previous MRI studies have indicated that NM signal intensity in the SN are decreased even in the early stages of PD (22, 26). NM has a high binding affinity for iron. However, Reimao et al. reported that there is no significant correlation between NM and iron content in the SN (26). A recent review demonstrated that the NM is not only directly involved in reactive oxygen species (ROS) reduction but also in Ca^2+^ homeostasis, with NM loss leading to the death of dopaminergic neurons (27). Consistent with our quantitative results, a previous analysis study (14) demonstrated that NM levels in the SN are not significantly decreased in patients with ET. Indeed, only 20% of patients with ET obtained scores of 1. All others obtained scores of 0. In contrast, 39.7 and 48.5% of patients with PD obtained scores of 1 and 2, respectively. These results may provide evidence against a possible pathogenic link between PD and ET.

Consistent with the findings of previous reports (17), our results indicated that N1 was absent on QSM images in 79.4% of patients with PD. While NM is known to participate in intra-cellular iron metabolism, loss of nigrosome signals may be related to iron deposition in the brain (28). At present, QSM is the optimal imaging method for quantifying iron content in the brain *in vivo* (18, 20). Thus, our findings indicated that iron overload occurs in N1 in patients with PD. Previous studies have indicated that changes in N1 can aid in the differential diagnosis of PD (29), as they can be observed in both the early and late stages of the disease (17). Our data also demonstrated the presence of N1 in most patients with ET, in contrast to our findings for patients with PD.

To date, several studies have indicated that ET is likely to be a neurodegenerative disease, especially affecting cerebellar system proven by clinical, neuroimaging, and postmortem studies (30, 31). In addition, patients with ET exhibit a 4-fold higher risk of developing PD than those without ET (32). One MRI T2^*^-relaxometry study revealed that ET is associated with iron deposition in the SN (33). Another functional MRI study provided further evidence of neurodegeneration in patients with ET, reporting over-activation in the parietal cortex and dorsolateral prefrontal cortex (34). Mild abnormalities in striatal DATs have also been observed in patients with ET, along with a typical PD-like pattern of uptake loss (35). However, there is no loss of DAT binding over time in patients with ET, providing evidence against the neurodegeneration hypothesis (36). To our knowledge, our study is the first to report that the rate of N1 absence is significantly lower in patients with ET than in patients with PD, suggesting that ET is associated with a lower iron deposition than PD.

By combining our NM and N1 findings for patients with PD and ET, we developed diagnostic models for the differentiation of the two disorders. Molecular imaging techniques such as DAT imaging may further improve diagnostic accuracy. Novellino et al. reported that DAT-SPECT and MIBG scintigraphy findings are abnormal in patients with probable PD, while they are normal in patients with ET (37). Another study also reported that DAT imaging can be used to differentiate early-stage PD and ET with high sensitivity (84.4%) and specificity (96.2%) (38). However, this technology is not feasible for clinical application. Several transcranial sonography (TCS) studies have also aimed to distinguish patients with PD from those with ET (38–41), achieving moderate sensitivity and high specificity. Despite these results, recording failures due to an insufficient acoustic bone window may limit the use of TCS (42). A previous NM-MRI study reported sensitivity and specificity values of 67.7 and 93.3%, respectively, when high-signal areas in the SN were used to distinguish ET from PD (13). Because visual analysis is fast and more convenient for clinical applications, we performed visual analyses of NM and N1 to aid in the differential diagnosis of *de novo* PD and untreated ET. Sensitivity values were >79% for both biomarkers, while specificity values were equivalent to 80 and >90% for NM and N1, respectively. Despite the relatively good diagnostic ability of both visual assessments for NM and N1, further combining the two biomarkers may provide higher diagnostic accuracy in clinical practice for individual diagnosis. Indeed, the combination of both biomarkers achieved a sensitivity of 85.3% and specificity of 92%—values higher than those for each biomarker.

The present study possesses some limitations of note. First, the number of participants was relatively small, especially the number of patients with ET, and all participants were recruited from clinical settings. Therefore, replication in larger population-dwelling samples is warranted to confirm the effectiveness of the diagnostic models used in our study. Second, NM-MRI acquisition times were relatively long in our study, which many patients may be unable to tolerate. However, a recent study introduced a three-dimensional NM-MRI sequence that took only slightly more than 4 min, which may facilitate clinical application of this method (43). Third, the analysis in our study was qualitative, which may be more suitable for clinical application, rather than quantitative. However, QSM can be used to quantify iron content (20), while NM content can be quantified on NM-MRI based on volume/width (22, 44). Further studies should therefore focus on the quantitative differences between PD and ET. Notably, the patients recruited in this study were all drug naïve, which eliminated the undefined confounding factors associated with medication use (45).

In conclusion, our findings indicated that visual analyses combing NM and N1 may represent a diagnostic biomarker for the differentiation of tremor disorders. Furthermore, our results suggest that iron deposition is greater in patients with PD than in those with ET.

## Author Contributions

All authors have carefully reviewed this manuscript. In this study, each author has contributed significantly. LJ, YL, CW, YiZ, XC, ZH, ML, CZ, and GF were responsible for collecting and confirming clinical data. JW, DL, KL, MZ, and YoZ were responsible for the MRI study. CZ designed the study and wrote the manuscript.

### Conflict of Interest Statement

CZ holds shares of Shanghai Rixin Biotech Co., Ltd., which focuses on the development of new drugs against Alzheimer's disease. The remaining authors declare that the research was conducted in the absence of any commercial or financial relationships that could be construed as a potential conflict of interest.

## References

[1] Louis ED Ferreira JJ. How common is the most common adult movement disorder? Update on the worldwide prevalence of essential tremor. Mov Disord. (2010) 25:534–41. 10.1002/mds.22838 20175185

[2] Kwon KY Lee HM Lee SM Kang SH Koh SB. Comparison of motor and non-motor features between essential tremor and tremor dominant Parkinson's disease. J Neurol Sci. (2016) 361:34–8. 10.1016/j.jns.2015.12.016 26810513

[3] Thenganatt MA Jankovic J. The relationship between essential tremor and Parkinson's disease. Parkinsonism Relat Disord. (2016) 22(Suppl 1):S162–5. 10.1016/j.parkreldis.2015.09.032 26522219

[4] Postuma RB Berg D Stern M Poewe W Olanow CW Oertel W. MDS clinical diagnostic criteria for Parkinson's disease. Mov Disord. (2015) 30:1591–601. 10.1002/mds.26424 26474316

[5] Heim B Krismer F De Marzi R Seppi K. Magnetic resonance imaging for the diagnosis of Parkinson's disease. J Neural Transm. (2017) 124:915–64. 10.1007/s00702-017-1717-8 28378231 PMC5514207

[6] Kassubek J. MRI-based neuroimaging: atypical parkinsonisms and other movement disorders. Curr Opin Neurol. (2018) 31:425–30. 10.1097/WCO.0000000000000578 29952835

[7] Lehericy S Vaillancourt DE Seppi K Monchi O Rektorova I Antonini A. The role of high-field magnetic resonance imaging in parkinsonian disorders: pushing the boundaries forward. Mov Disord. (2017) 32:510–25. 10.1002/mds.26968 28370449

[8] Kalia LV Lang AE. Parkinson's disease. Lancet (2015) 386:896–912. 10.1016/S0140-6736(14)61393-3 25904081

[9] Shill HA Adler CH Beach TG. Pathology in essential tremor. Parkinsonism Relat Disord. (2012) 18(Suppl 1):S135–7. 10.1016/S1353-8020(11)70042-6 22166412

[10] Sasaki M Shibata E Tohyama K Takahashi J Otsuka K Tsuchiya K. Neuromelanin magnetic resonance imaging of locus ceruleus and substantia nigra in Parkinson's disease. Neuroreport (2006) 17:1215–8. 10.1097/01.wnr.0000227984.84927.a7 16837857

[11] Matsuura K Maeda M Yata K Ichiba Y Yamaguchi T Kanamaru K. Neuromelanin magnetic resonance imaging in Parkinson's disease and multiple system atrophy. Eur Neurol. (2013) 70:70–7. 10.1159/000350291 23796701

[12] Reimao S Pita Lobo P Neutel D Correia Guedes L Coelho M Rosa MM. Substantia nigra neuromelanin magnetic resonance imaging in *de novo* Parkinson's disease patients. Eur J Neurol. (2015) 22:540–6. 10.1111/ene.12613 25534480

[13] Reimao S Pita Lobo P Neutel D Guedes LC Coelho M Rosa MM. Substantia nigra neuromelanin-MR imaging differentiates essential tremor from Parkinson's disease. Mov Disord. (2015) 30:953–9. 10.1002/mds.26182 25758364

[14] Wang J Huang Z Li Y Ye F Wang C Zhang Y. Neuromelanin-sensitive MRI of the substantia nigra: an imaging biomarker to differentiate essential tremor from tremor-dominant Parkinson's disease. Parkinsonism Relat Disord. (2018) S1353-8020(18)30309-2. 10.1016/j.parkreldis.2018.07.007 30037690

[15] Damier P Hirsch EC Agid Y Graybiel AM. The substantia nigra of the human brain. II. Patterns of loss of dopamine-containing neurons in Parkinson's disease. Brain (1999) 122(Pt 8):1437–48. 10430830 10.1093/brain/122.8.1437

[16] Lehericy S Bardinet E Poupon C Vidailhet M Francois C. 7 Tesla magnetic resonance imaging: a closer look at substantia nigra anatomy in Parkinson's disease. Mov Disord. (2014) 29:1574–81. 10.1002/mds.26043 25308960

[17] Schwarz ST Afzal M Morgan PS Bajaj N Gowland PA Auer DP. The 'swallow tail' appearance of the healthy nigrosome - a new accurate test of Parkinson's disease: a case-control and retrospective cross-sectional MRI study at 3T. PLoS ONE (2014) 9:e93814. 10.1371/journal.pone.0093814 24710392 PMC3977922

[18] Bilgic B Pfefferbaum A Rohlfing T Sullivan EV Adalsteinsson E. MRI estimates of brain iron concentration in normal aging using quantitative susceptibility mapping. Neuroimage (2012) 59:2625–35. 10.1016/j.neuroimage.2011.08.077 21925274 PMC3254708

[19] Liu T Xu W Spincemaille P Avestimehr AS Wang Y. Accuracy of the morphology enabled dipole inversion (MEDI) algorithm for quantitative susceptibility mapping in MRI. IEEE Trans Med Imaging (2012) 31:816–24. 10.1109/TMI.2011.2182523 22231170 PMC3613569

[20] He N Ling H Ding B Huang J Zhang Y Zhang Z. Region-specific disturbed iron distribution in early idiopathic Parkinson's disease measured by quantitative susceptibility mapping. Hum Brain Mapp. (2015) 36:4407–20. 10.1002/hbm.22928 26249218 PMC6869507

[21] Deuschl G Bain P Brin M. Consensus statement of the Movement Disorder Society on Tremor. *Ad hoc* scientific committee. Mov Disord. (1998) 13(Suppl 3):2–23. 9827589 10.1002/mds.870131303

[22] Wang J Li Y Huang Z Wan W Zhang Y Wang C. Neuromelanin-sensitive magnetic resonance imaging features of the substantia nigra and locus coeruleus in *de novo* Parkinson's disease and its phenotypes. Eur J Neurol. (2018) 25:949–e973. 10.1111/ene.13628 29520900

[23] Pyatigorskaya N Magnin B Mongin M Yahia-Cherif L Valabregue R Arnaldi D. Comparative study of MRI biomarkers in the substantia nigra to discriminate idiopathic Parkinson disease. AJNR Am J Neuroradiol. (2018) 39:1460–7. 10.3174/ajnr.A5702 29954816 PMC7410545

[24] Benito-Leon J Louis ED. Clinical update: diagnosis and treatment of essential tremor. Lancet (2007) 369:1152–4. 10.1016/S0140-6736(07)60544-3 17416247

[25] Martin-Bastida A Pietracupa S Piccini P. Neuromelanin in parkinsonian disorders: an update. Int J Neurosci. (2017) 127:1116–23. 10.1080/00207454.2017.1325883 28460588

[26] Reimao S Ferreira S Nunes RG Pita Lobo P Neutel D Abreu D. Magnetic resonance correlation of iron content with neuromelanin in the substantia nigra of early-stage Parkinson's disease. Eur J Neurol. (2016) 23:368–74. 10.1111/ene.12838 26518135

[27] Knorle R. Neuromelanin in Parkinson's Disease: from Fenton Reaction to Calcium Signaling. Neurotox Res. (2018) 33:515–22. 10.1007/s12640-017-9804-z 28879408

[28] Massey LA Miranda MA Al-Helli O Parkes HG Thornton JS So PW. 9.4T MR microscopy of the substantia nigra with pathological validation in controls and disease. Neuroimage Clin. (2017) 13:154–63. 10.1016/j.nicl.2016.11.015 27981030 PMC5144755

[29] Mahlknecht P Krismer F Poewe W Seppi K. Meta-analysis of dorsolateral nigral hyperintensity on magnetic resonance imaging as a marker for Parkinson's disease. Mov Disord. (2017) 32:619–23. 10.1002/mds.26932 28151553

[30] Novellino F Nicoletti G Cherubini A Caligiuri ME Nistico R Salsone M. Cerebellar involvement in essential tremor with and without resting tremor: a diffusion tensor imaging study. Parkinsonism Relat Disord. (2016) 27:61–6. 10.1016/j.parkreldis.2016.03.022 27050442

[31] Louis ED. Essential tremor and the cerebellum. Handb Clin Neurol. (2018) 155:245–58. 10.1016/B978-0-444-64189-2.00016-0 29891062

[32] Benito-Leon J Louis ED Bermejo-Pareja F Neurological Disorders in Central Spain Study G. Risk of incident Parkinson's disease and parkinsonism in essential tremor: a population based study. J Neurol Neurosurg Psychiatry (2009) 80:423–5. 10.1136/jnnp.2008.147223 19289477

[33] Novellino F Cherubini A Chiriaco C Morelli M Salsone M Arabia G. Brain iron deposition in essential tremor: a quantitative 3-Tesla magnetic resonance imaging study. Mov Disord. (2013) 28:196–200. 10.1002/mds.25263 23238868

[34] Cerasa A Passamonti L Novellino F Salsone M Gioia MC Morelli M. Fronto-parietal overactivation in patients with essential tremor during Stroop task. Neuroreport (2010) 21:148–51. 10.1097/WNR.0b013e328335b42c 20010442

[35] Isaias IU Canesi M Benti R Gerundini P Cilia R Pezzoli G. Striatal dopamine transporter abnormalities in patients with essential tremor. Nucl Med Commun. (2008) 29:349–53. 10.1097/MNM.0b013e3282f4d307 18317299

[36] Isaias IU Marotta G Hirano S Canesi M Benti R Righini A. Imaging essential tremor. Mov Disord. (2010) 25:679–86. 10.1002/mds.22870 20437537

[37] Novellino F Arabia G Bagnato A Cascini GL Salsone M Nicoletti G. Combined use of DAT-SPECT and cardiac MIBG scintigraphy in mixed tremors. Mov Disord. (2009) 24:2242–8. 10.1002/mds.22771 19795467

[38] Jesus-Ribeiro J Freire A Sargento-Freitas J Sousa M Silva F Moreira F. Transcranial sonography and DaTSCAN in early stage parkinson's disease and essential tremor. Eur Neurol. (2016) 76:252–5. 10.1159/000452216 27750247

[39] Stockner H Sojer MKKS Mueller J Wenning GK Schmidauer C. Midbrain sonography in patients with essential tremor. Mov Disord. (2007) 22:414–7. 10.1002/mds.21344 17226854

[40] Budisic M Trkanjec Z Bosnjak J Lovrencic-Huzjan A Vukovic V Demarin V. Distinguishing Parkinson's disease and essential tremor with transcranial sonography. Acta Neurol Scand. (2009) 119:17–21. 10.1111/j.1600-0404.2008.01056.x 18549415

[41] Stockner H Wurster I. Transcranial sonography in essential tremor. Int Rev Neurobiol. (2010) 90:189–97. 10.1016/S0074-7742(10)90014-7 20692503

[42] Okawa M Miwa H Kajimoto Y Hama K Morita S Nakanishi I. Transcranial sonography of the substantia nigra in Japanese patients with Parkinson's disease or atypical parkinsonism: clinical potential and limitations. Intern Med. (2007) 46:1527–31. 10.2169/internalmedicine.46.0271 17878638

[43] Prasad S Stezin A Lenka A George L Saini J Yadav R. Three-dimensional neuromelanin-sensitive magnetic resonance imaging of the substantia nigra in Parkinson's disease. Eur J Neurol. (2018) 25:680–6. 10.1111/ene.13573 29341412

[44] Chen X Huddleston DE Langley J Ahn S Barnum CJ Factor SA. Simultaneous imaging of locus coeruleus and substantia nigra with a quantitative neuromelanin MRI approach. Magn Reson Imaging (2014) 32:1301–6. 10.1016/j.mri.2014.07.003 25086330

[45] Fahn S Oakes D Shoulson I Kieburtz K Rudolph A Lang A. Levodopa and the progression of Parkinson's disease. N Engl J Med. (2004) 351:2498–508. 10.1056/NEJMoa033447 15590952

